# HuoXueTongFu Formula Alleviates Intraperitoneal Adhesion by Regulating Macrophage Polarization and the SOCS/JAK2/STAT/PPAR-*γ* Signalling Pathway

**DOI:** 10.1155/2019/1769374

**Published:** 2019-10-21

**Authors:** Min Zhao, Yao-Yao Bian, Li-Li Yang, Yan-Qi Chen, Ya-Jie Wang, Yan-Ting Ma, Yu-Qiong Pei, Wen-Lin Li, Li Zeng

**Affiliations:** ^1^The First School of Clinical Medicine, Nanjing University of Chinese Medicine, Nanjing 210046, China; ^2^School of Nursing, Nanjing University of Chinese Medicine, Nanjing 210046, China; ^3^The Second School of Clinical Medicine, Nanjing University of Chinese Medicine, Nanjing 210046, China; ^4^School of Pharmacy, Nanjing University of Chinese Medicine, Nanjing 210046, China; ^5^Library, Nanjing University of Chinese Medicine, Nanjing 210046, China

## Abstract

Intraperitoneal adhesion is a common complication after abdominal surgery, which seriously affects the quality of life of patients. HuoXueTongFu Formula (HXTF) plays an important role in the prevention and treatment of intraperitoneal adhesions. However, the molecular-related mechanisms are still not fully known. In this study, the model of Intrapetitoneal adhesion was established by cecum abrasion and treated with HXTF for one week. RAW264.7 cells were given LPS, IFN-*γ*, IL-4, HXTF-medicated serum, and PPAR-*γ* agonist/antagonist, respectively. Histopathology, flow cytometry, ELISA, real-time PCR, and Western blotting were used to further detect the related protein, M1/M2 polarization tendency, and PPAR-*γ* nuclear translocation. The deposition of collagen fibres reduced in the local area of rats after the operation with HXTF treatment. Similar to IL-4, HXTF induced a tendency for macrophages to polarize toward M2 and promoted peroxisome proliferator-activated receptor-gamma (PPAR-*γ*) nuclear translocation. Furthermore, the use of HXTF and PPAR-*γ* agonists downregulated macrophage M1 polarization-related factors IL-1, IL-6, and TNF-alpha and upregulated M2 polarization-related factors IL-4, IL-10, and TGF-beta 1. Meanwhile, the use of HXTF and PPAR-*γ* agonists downregulated the SOCS3/JAK2/STAT1 pathway and activated the SOCS1/STAT6/PPAR-*γ* pathway. These results show that HXTF may reduce intraperitoneal adhesion by inducing macrophage M2 polarization and regulating the SOCS/JAK2/STAT/PPAR-*γ* pathway.

## 1. Introduction

Intraperitoneal adhesions have been reported to occur after 93-100% of upper abdominal laparotomy and 67-93% of lower abdominal laparotomy [1], but the location, severity, time, and type of symptoms are different [2]. They exist in the form of tiny vascularized membranes to actual connective tissue bridges that may contain blood vessels and nerve structures or direct bonding contacts between adjacent organs. This “bridge” may lead to abdominal pain, intestinal obstruction, infertility, and difficulty in reoperation [3]. Retrospective studies have found that intestinal obstruction is a major complication of intraperitoneal adhesions, and it is involved in 32% of acute intestinal obstruction and 65-75% of small intestinal obstruction [1]. In Sweden, the cost of treatment for small-bowel obstruction associated with intraperitoneal adhesions is estimated at 40-60 million euros/year [4]. In the United States, as early as 1994, the cost associated with adhesiolysis had reached $1.3 billion [5]. Therefore, it is necessary to prevent and treat abdominal adhesions, whether for the health of patients or for relieving the burden of medical care.

Cytokines released by infiltration of inflammatory cells and oxidative stress are considered triggering mechanisms and initial steps leading to adhesion formation [6–8]. Macrophages are involved in the occurrence, progression, and digestion of inflammation and fibrin deposition. Macrophages are a group of heterogeneous cells with great plasticity. Their phenotype and function are regulated by the surrounding microenvironment, and their functional plasticity is closely related to polarization activation [9]. It is generally believed that lipopolysaccharide (LPS) alone or in combination with Th1 cytokines (such as IFN-*γ* and GM-CSF) induces macrophage activation into M1-type macrophages (M1), which have proinflammatory properties and activate Toll-like receptor 4 signalling. Th2 cytokines (such as IL-4 and IL-13) induce macrophage activation into M2 macrophages (M2), which have anti-inflammatory and immunoregulatory functions [10, 11]. It has been found that the levels of M1 phenotype-related proinflammatory cytokines such as TNF-*α*, IL-6, and IFN-*γ* are significantly increased in adhesion tissues, while the cytokines and markers associated with the M2 phenotype also changed, such as decreased expression of CD206, YM1, and Arg-1 [12]. Up-down or parallel relationship of SOCSs/JAK/STATs/PPAR-*γ* coordinates the polarization activation of macrophages [13, 14].

Previous animal experiments showed that HuoXueTongFu Formula (HXTF) could play an antiadhesion role through an intestinal mucosal immune barrier [15] and oxidative stress [16]. According to the significant clinical effect of HXTF on intraperitoneal adhesions [17] and the basis of experimental research, we established a RAW264.7 macrophage inflammation model and rat intraperitoneal adhesion model to observe whether HXTF affects inflammation responses by regulating macrophage polarization and the SOCS/JAK/STAT/PPAR-*γ* pathway.

## 2. Materials and Methods

### 2.1. Reagents

Fluvastatin capsules were purchased from Novartis Pharmaceutical Co., Ltd. (Beijing, China). A Masson staining kit was purchased from Leagene Biotechnology Co., Ltd. (Beijing, China). A hematoxylin-eosin staining kit was purchased from Servicebio Technology Co., Ltd. (Wuhan, China). Cell Counting Kit-8 (CCK-8) was obtained from Fcmacs Biotech Co., Ltd. (Nanjing, China). A BCA protein assay kit was purchased from Beyotime Biotechnology Co., Ltd. (Shanghai, China). Rosiglitazone (RSG, selective PPAR-*γ* agonist) and T0070907 (selective PPAR-*γ* antagonist) were obtained from Selleck Chemicals (Houston, USA). LPS was purchased from Sigma Chemical (St. Louis, USA). Recombinant rat IL-4 and INF-*γ* were purchased from PeproTech Inc. (New Jersey, USA). ELISA kits (IL-1, IL-6, TNF-*α*, IL4, IL-10, IL-13, and TGF-*β*1) were obtained from JinYiBai Biological Technology (Nanjing, China). The antibodies (JAK2, STAT6, and p-STAT1) were purchased from Santa Cruz Biotechnology (California, USA). The antibodies (PPAR-*γ*, STAT1, and p-PPAR-*γ*) were purchased from Bioss (Beijing, China). The antibodies (SOCS1, SOCS3, p-JAK2, and p-STAT6) were purchased from Affinity Biosciences. APC-anti-mouse CD86 was purchased from BioLegend (San Diego, USA). PE-Cy7-anti-mouse CD206 and Intracellular Fixation & Permeabilization set were purchased from Thermo Fisher Scientific (Massachusetts, USA).

### 2.2. HXTF Preparation

The HXTF is composed of six crude herbs: *Chinese rhubarb*, *peach kernel*, *Corydalis yanhusuo*, *radish seed*, *Glauber's salt*, and *safflower* at a ratio of 5 : 5 : 5 : 5 : 5 : 3 (details about the herbs could be seen in Table 1) (Chinese rhubarb, Semen Persicae, Rhizoma Corydalis, Semen Raphani, Natrii Sulfas, and Flos Carthami). The medicinal materials were purchased from Jiangsu Province Hospital of Chinese Medicine and authenticated by the Processing Laboratory of the Nanjing University of Chinese Medicine as genuine medicinal materials with quality standards. Radix et Rhizoma Rhei, Semen Persicae, Rhizoma Corydalis, and Semen Raphani were extracted twice by reflux with 70% ethanol 15 times the total weight of the decoction pieces, 2 hours each time, and filtered and the extracts were combined and filtered, and the ethanol was evaporated under vacuum and concentrated to no alcohol odor. Then, the residue and Flos Carthami were combined and decocted three times with water 20 times the total weight of the decoction pieces, one hour each time, filtered, and combined with water decoction. The filtrate was concentrated to the relative density of 1.09-1.11, cooled, added ethanol to 50% alcohol content, stirred, and stored for 48 hours, and the supernatant was taken and then added Natrii Sulfas, concentrated to the relative density of 1.12-1.15, and set aside.

### 2.3. Preparation of HXTF-Medicated Serum

Sprague-Dawley (SD) rats were randomly divided into HXTF (*n* = 8) and vehicle control (*n* = 15) groups. Rats in the HXTF group received HXTF (10.44 g/kg, p.o.) twice a day for three days, whereas the control group received physiological saline. One hour after the last administration, the rats were intraperitoneally anesthetized with pentobarbital (4 mg/100 g), and blood was taken from the abdominal aorta and centrifuged. The supernatant was inactivated at 56°C for 30 min, filtered and sterilized through the 0.22 *μ*m filter, and stored at -80°C for use.

### 2.4. The Chemical Analysis of HXTF by HPLC

The operation was performed using a 1260 high-performance liquid chromatograph (Agilent, USA) and an OpenLAB CDS chromatography workstation (Agilent, USA). The Elite SinoChrom BP C18 column (4.6 mm × 250 mm, 5 *μ*m) was used. Mobile phase A and mobile phase B are methanol and 1% glacial acetic acid solution, respectively. The gradient was eluted as follows: 0-5 min, 10-15% A; 5-45 min, 15-55% A; 45-75 min, 55-75% A; 75-90 min, 75-90% A; 90-95 min, 90-95% A; and 95-98 min, 95-10% A. The flow rate is 1.0 mL/min. The wavelength is 254 nm. The injection volume is 10 *μ*L. The column temperature is 30°C.

### 2.5. Animals and Surgical Procedure

Male SD rats, weighing 250 ± 20 g, were purchased from Shanghai Jiesijie Laboratory Animal Co., Ltd. (Shanghai, China) and housed in standard cages in a 12 h light/dark cycle. The room temperature is 18-25°C, and the relative humidity is 65%-70%. All experiments were conducted by the guidelines of current ethical regulations for institutional animal care and use in the Nanjing University of Chinese Medicine. All animal experiments were made to minimize suffering and reduce the number of animals used. The experiment was approved by the Ethics Committee of Nanjing University of Chinese Medicine (ACU171112, 2017-11-22).

Forty SD rats were randomly divided into four groups: (1) sham group (*n* = 10), (2) intraperitoneal adhesion group (IA, *n* = 10), (3) HXTF+IA group (10.44 g/kg, *n* = 10), and (4) fluvastatin+IA group (FS+IA, 10 mg/kg, *n* = 10). The administration group was given orally once a day within 7 days after operation.

Surgical intervention was performed on 40 rats. Surgical intervention was performed under aseptic conditions, and the surgical instruments were sterilized using a steam autoclave (Tomy, Japan) the night before the operation. Before the surgical intervention, the rats were fasted for 12 hours and were free to drink water. Rats were anesthetized by intraperitoneal injection with pentobarbital. The abdominal hair was shaved, the supine position was fixed, and the abdomen was disinfected. As previously described [18], the cecum was found through a 1.5 cm incision at the anterior midline of the lower abdomen. The cecum serosa layer was rubbed repeatedly with a sickle until punctate bleeding appeared on the surface of the cecum, resulting in a wound of about 2.0 cm × 1.5 cm. The cecum was exposed to air for 5 min, then incorporated into the abdominal cavity and sutured layer by layer with 3-0 sterile silk. In the sham operation group, the abdominal cavity was exposed to air 5 min after open surgery without rubbing. All rats were sacrificed on the seventh day after surgery, and a U-shaped incision was performed in the lower abdomen.

### 2.6. Macroscopic Evaluation

Two experimentally unrelated individuals performed objective adhesion evaluation and scoring. The adhesions were graded in a blinded fashion using the Kennedy method, as described in Table 2 [19, 20].

### 2.7. Cell Culture and Administration

Murine macrophage RAW264.7 cells were kindly provided by Stem Cell Bank, Chinese Academy of Sciences (Shanghai, China). The cells were cultured in Dulbecco's modified Eagle medium (DMEM) (Gibco, USA) containing 10% fetal bovine serum (Gibco, USA), 100 U/mL penicillin, and 100 *μ*g/mL streptomycin (Gibco, USA) at 37°C in a humidified incubator containing 5% CO_2_ and 95% air.

All cells were in a logarithmic growth phase when they were intervened. RAW264.7 cells were induced to polarize with DMEM solution and LPS (100 ng/mL)+IFN-*γ* (20 ng/mL) or IL-4 (20 ng/mL) for 12 hours. PPAR-*γ* agonists (rosiglitazone, 1 *μ*mol) or antagonists (T0070907, 1 *μ*mol) were precultured for 3 hours, then combined with LPS+IFN-*γ*, IL-4, and HXTF-medicated serum for 12 hours.

### 2.8. Cell Viability

The cell viability was detected using CCK-8. 100 *μ*L of RAW264.7 cell suspension (2 × 10^4^ cells/well) was seeded into 96-well plates and attached for 8 hours. Then, the supernatant was removed and cultured in serum-free medium for 12 hours. HXTF-medicated serum was then added at the various concentrations for 24 hours. Following treatment, 10 *μ*L of CCK-8 solution was added to each pore and incubated for 2 hours, and the absorbance was measured at 450 nm.

### 2.9. Histopathological Examination

The cecum specimens were collected and treated as follows: cecum tissue was placed in 10% formaldehyde for 48 h, rinsed with water, and dehydrated using different concentrations of ethanol, followed by embedding in paraffin, sectioning (4 *μ*m thick), heating, and dewaxing. The sections were stained with Masson trichrome (MT) to assess the degree of fibrosis, and sections were stained with hematoxylin-eosin (HE) to evaluate the inflammation.

### 2.10. Western Blot Analysis

The caecum and adhesive tissues of rats and RAW264.7 cells were lysed. The protein concentrations were determined by the BCA protein assay (Thermo Scientific) according to the manufacturer's instructions. The same amount of protein was loaded and separated by sodium dodecyl sulfate-polyacrylamide gel electrophoresis (SDS-PAGE) (Bio-Rad, Hercules, USA) and then transferred to a polyvinylidene fluoride (PVDF) membrane. According to the molecule, the size-cut membrane was blocked with 5% skim milk for 1 hour at room temperature and then incubated with the primary antibody (diluted antibody specification corresponding to antibody incubation) at 4°C overnight. *β*-Actin was used as an internal protein. The membrane was then incubated with the corresponding secondary antibody for 80 minutes at room temperature. Finally, imaging was performed by using a fully automated chemiluminescent gel imaging system (Bio-Rad, Hercules, USA).

### 2.11. Real-Time PCR

Total RNA was extracted from the cecum and adhesion tissue of rats, using TRIzol reagent (Servicebio, China). RNA concentration and purity were measured using Nanodrop 2000, and the overconcentrated RNA was diluted in an appropriate ratio to a final concentration of 200 ng/*μ*L. cDNA was synthesized from 2 *μ*g of total RNA using the RevertAid First Strand cDNA Synthesis Kit (Thermo, USA). For real-time PCR, the 0.2 mL PCR tube was prepared to prepare the following reaction system, and all reactions were performed in triplicate: 2× SYBR qPCR Master Mix (Roche, Switzerland) (12.5 *μ*L), 7.5 *μ*M gene primer (2.0 *μ*L), reverse primer (2.5 *μ*L), and ddH2O (8.0 *μ*L). The reaction conditions were as follows: the predenaturation was 95°C for 10 min, followed by 40 cycles of 95°C for 15 s and 60°C for 60 s performed with ABI StepOnePlus (Applied Biosystems). All results were processed by the double-delta method (2^−*ΔΔ*Ct^). The primers (provided by Servicebio) are shown in Table 3.

### 2.12. Flow Cytometry

The 10^6^-10^8^ cells were collected into a dark-proof EP tube, and the volume was controlled at 100 *μ*L for the next experiment.

For the identification of M1/M2 polarization of RAW264.7 cells, CD86-APC (0.25 *μ*g/10^6^ cells) was incubated at 4°C for 30 min, then incubated with 100 *μ*L fixed buffer for 20-60 min at room temperature, and then incubated with CD206-PE-Cy7 (1.25 *μ*L/10^6^ cells) at 4°C for 30 min.

For the nuclear localization assay, the cells were incubated with 100 *μ*L fixation buffer for 20-60 min, then incubated with the PPAR-*γ* antibody (1 : 500) for 2 h, and then incubated with the FITC fluorescent secondary antibody (1 : 1000) for 1 h. All operations are performed at room temperature.

All the above experiments were detected by flow cytometry (Merck Millipore, USA) and analyzed with IDEAS software.

### 2.13. Statistical Analysis

All statistical analyses were performed using SPSS software. Parametric and nonparametric tests were used based on the normality and distribution of the data. Normally distributed continuous data were expressed as the mean ± standard error (SE). Nonparametric data were expressed as median ± interquartile range (IQR). One-way ANOVA was used for normally distributed continuous data, and the Kruskal-Wallis ANOVA test was used for nonnormally distributed continuous data. The level of statistical significance was established as *p* < 0.05.

## 3. Results

### 3.1. HPLC Analysis of the Extract

**Figure 1:**
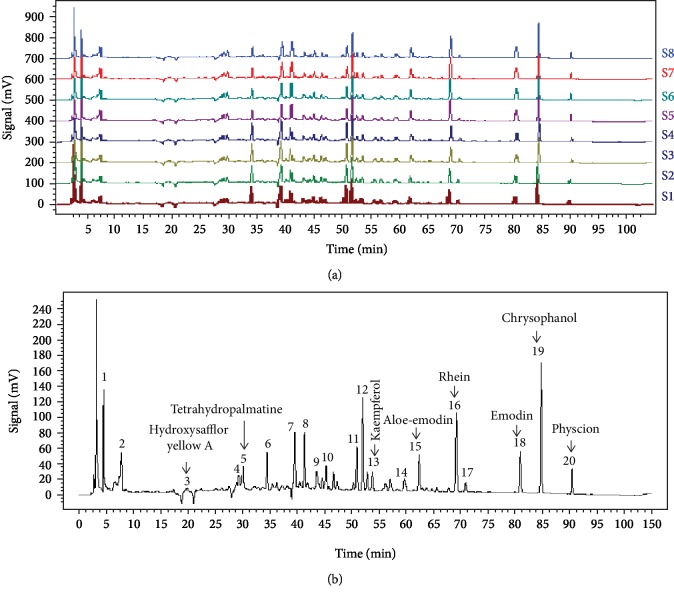
Fingerprints of HuoXueTongFu Formula (HXTF): (a) chromatographic fingerprints of eight samples of HXTF (S1–S8) and (b) the typical chromatographic fingerprint for HXTF. The 8 common peaks are labeled.

Eight samples of HXTF were used to develop the standard fingerprints (Figure 1(a)). Peaks presented in all 8 samples were defined as “common peaks.” As a result, 20 characteristic peaks shown in the fingerprint chromatogram were assigned as common peaks (Figure 1(b)). Eight peaks were identified, and their retention time (RTS) and ultraviolet absorption spectra were compared to identify the following components: hydroxysafflor yellow A, tetrahydropalmatine, kaempferol, aloe-emodin, rhein, emodin, chrysophanol, and physcion.

### 3.2. The Effect of HXTF on Cell Viability

To assess the effect of HXTF on RAW 264.7 cells, we measured cell viability using CCK-8. As shown in [Supplementary-material supplementary-material-1], cell viability was not affected by HXTF within 24 hours at 10% of HXTF-medicated serum.

### 3.3. HXTF Affects the Polarization of Macrophages

**Figure 2:**
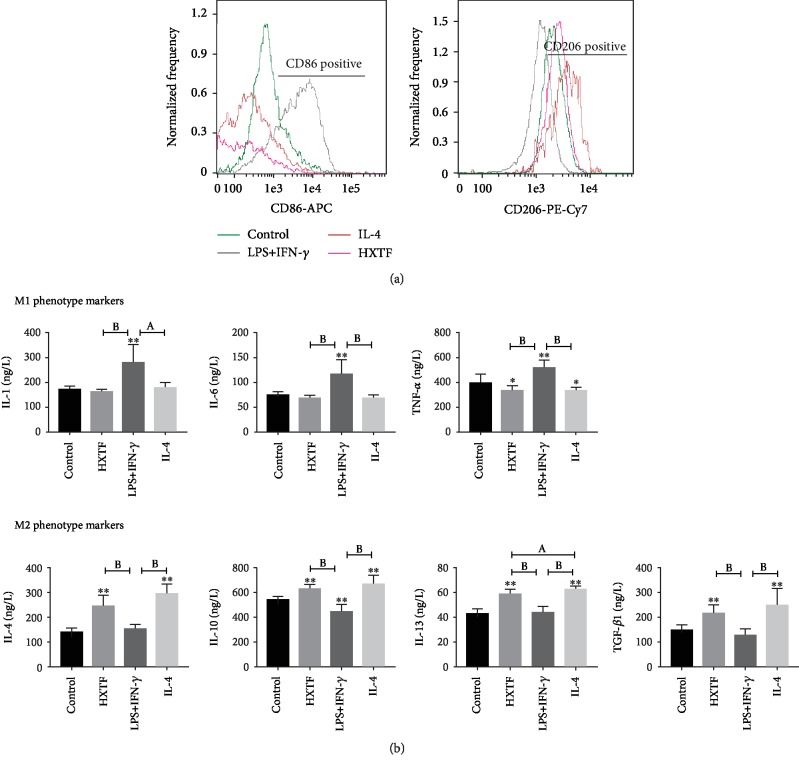
Effects of HuoXueTongFu Formula (HXTF) on macrophage polarization. RAW264.7 macrophages were incubated during 12 h with the control DMEM solution, LPS+IFN-*γ*, IL-4, and HXTF, respectively. (a) Flow cytometry analysis of M1/M2 marker CD86 and CD206 expression. (b) ELISA analysis of M1/M2 gene marker expression on macrophages. All values are expressed as mean ± S.E. (*n* = 3 experiments). ^∗^*p* < 0.05, ^∗∗^*p* < 0.01, ^a^*p* < 0.05, and ^b^*p* < 0.01, comparison of the designated two groups.

CD86 and CD206 are markers of the macrophage M1 and M2 phenotypes, respectively. Macrophages treated with HXTF showed a tendency of M2 polarization. Flow cytometry showed that M1 expression in the HXTF group was significantly lower than that in the LPS+IFN-*γ* group, and the M2 expression level was similar to that in the IL-4 group (Figure 2(a)). At the same time, HXTF also enhanced the expression of some M2 markers, including IL-4, IL-10, and IL-13 (Figure 2(b)).

### 3.4. HXTF Reduces the M1 Polarization Induced by LPS+IFN-*γ*

**Figure 3:**
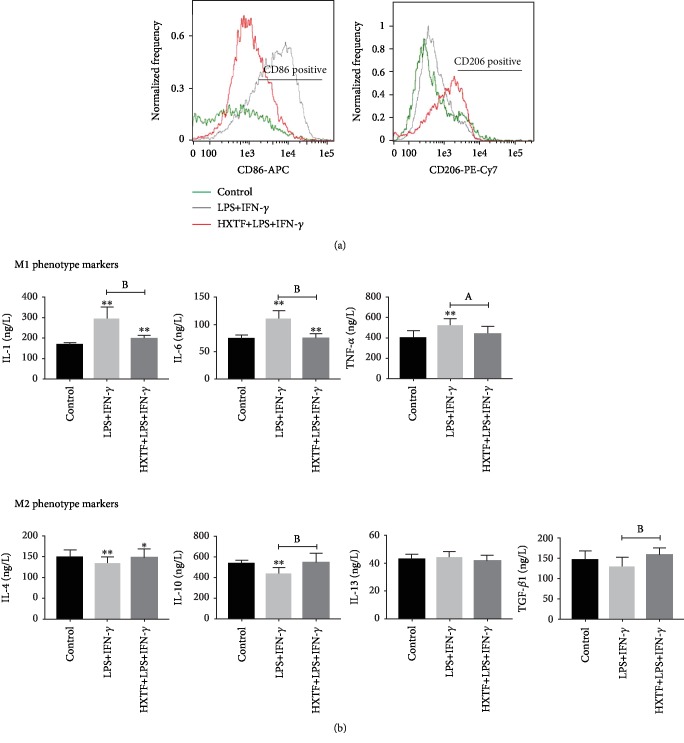
Effect of HuoXueTongFu Formula (HXTF) on M1 polarization induced by LPS+IFN-*γ*. RAW264.7 macrophages were incubated during 12 h with the control DMEM solution and LPS+IFN-*γ*, respectively. Meanwhile, RAW264.7 cells were preincubated with LPS+IFN-*γ* for 3 h, followed by combined treatment with HXTF for 12 h. (a) Flow cytometry analysis of M1/M2 marker CD86 and CD206 expression. (b) ELISA analysis of M1/M2 gene marker expression on macrophages. All values are expressed as mean ± S.E. (*n* = 3 experiments). ^∗^*p* < 0.05, ^∗∗^*p* < 0.01, ^a^*p* < 0.05, and ^b^*p* < 0.01, comparison of the designated two groups.

Macrophages treated with LPS+IFN-*γ* showed an obvious tendency to polarize toward M1 (Figure 3(a)). The expression of M1-related markers increased, including IL-1, IL-6, and TNF-*α*, while the expression of M2-related markers decreased, including IL-4 and IL-10 (Figure 3(b)). Compared with the LPS+IFN-*γ* group, macrophages treated with LPS+IFN-*γ*+HXTF showed a lower M1 polarization trend and decreased expression of the M1 markers (Figure 3).

### 3.5. HXTF Induces Macrophage Polarization through the SOCS/JAK2/STAT/PPAR-*γ* Pathway

**Figure 4:**
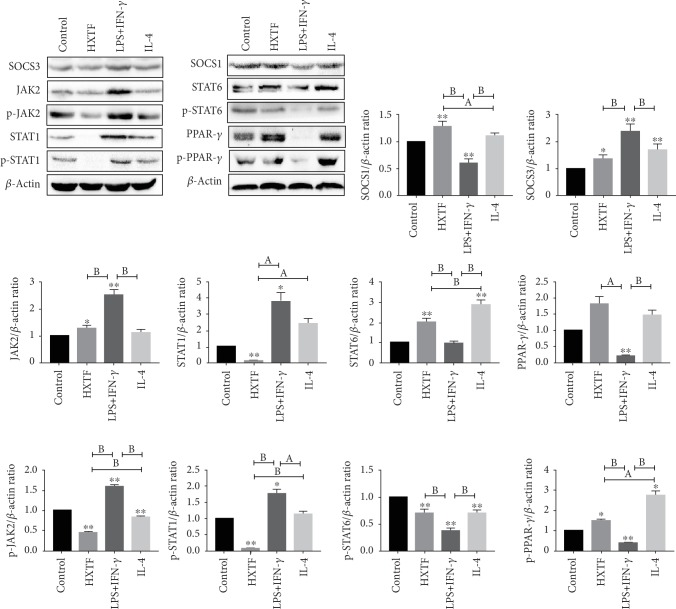
SOCS/JAK2/STAT/PPAR-*γ* pathway in HuoXueTongFu Formula- (HXTF-) induced macrophage polarization. RAW264.7 macrophages were incubated during 12 h with the control DMEM solution, LPS+IFN-*γ*, IL-4, and HXTF, respectively. Whole protein was extracted, and each protein expression was assayed by Western blotting. All values are expressed as mean ± S.E. (*n* = 3 experiments). ^∗^*p* < 0.05, ^∗∗^*p* < 0.01, ^a^*p* < 0.05, and ^b^*p* < 0.01, comparison of the designated two groups.

To elucidate the molecular mechanism of HXTF-induced macrophage polarization, we analyzed the SOCS/JAK2/STAT/PPAR-*γ* pathway-associated factors. We found that SOCS3, JAK2, STAT1, p-JAK2, and p-STAT1 were activated in the LPS+IFN-*γ* group and that SOCS1, STAT6, PPAR-*γ*, p-STAT6, and p-PPAR-*γ* were inhibited compared to the control group, whereas the IL-4 group showed the opposite effect on these factors (Figure 4). In contrast, the expression of related factors after HXTF treatment was similar to that after IL-4 treatment. In short, HXTF can activate SOCS1/STAT6/PPAR-*γ* of RAW264.7 macrophages in vitro, inhibit SOCS3/JAK2/STAT1, and promote the polarization of macrophages to M2.

### 3.6. Effect of HXTF on the Macrophage Phenotype and PPAR-*γ* Nuclear Translocation in Different Activities of PPAR-*γ*

**Figure 5:**
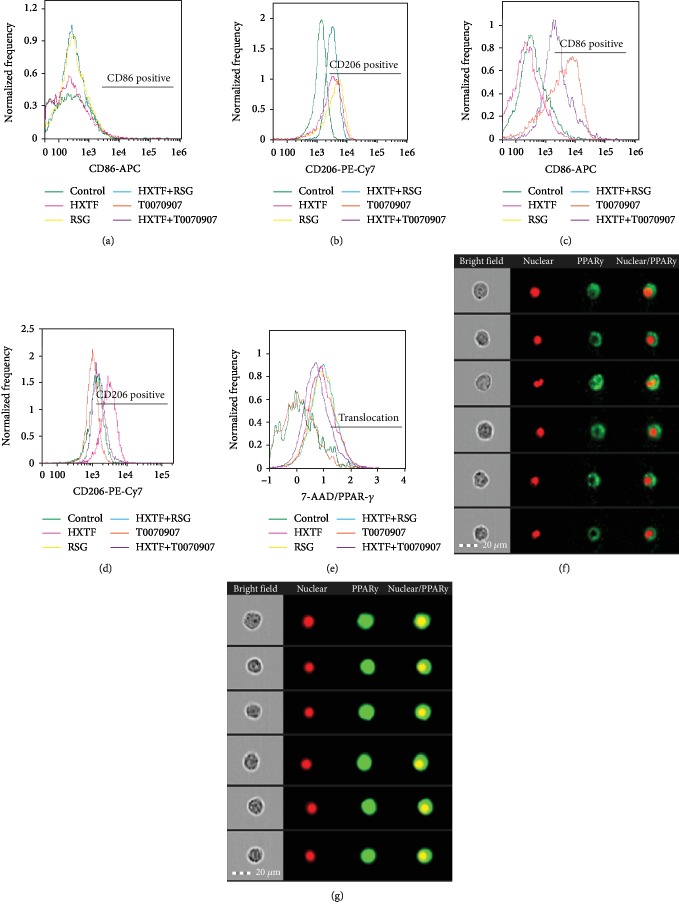
Modulation of PPAR-*γ* activity on macrophage polarization and PPAR-*γ* nuclear translocation. RAW264.7 cells were preincubated with either rosiglitazone (RSG) (1 *μ*mol/mL) or T0070907 (1 *μ*mol/mL) for 3 h, followed by combined treatment with/without HuoXueTongFu Formula (HXTF) for 12 h. (a, b) Flow cytometry analysis of M1/M2 marker CD86 and CD206 expression in control, HXTF, RSG, and HXTR+RSG group. (c, d) Flow cytometry analysis of M1/M2 marker CD86 and CD206 expression in control, HXTF, T0070907, and HXTF+T0070907 group. (e) Flow cytometry analysis of PPAR-*γ* nuclear translocation. (f) Typical cell figures of nuclear untranslocation. (g) Typical cell figures of nuclear translocation.

Numerous studies have shown that PPAR-*γ* is essential for the transformation of macrophages into the M2 phenotype [21, 22]. To confirm the effect of HXTF on macrophage phenotypic transformation in different activities of PPAR-*γ*, PPAR-*γ* agonist (rosiglitazone, RSG) and PPAR-*γ* antagonist (T0070907) were separately applied to the cell culture, with or without HXTF. Compared to the control group, RSG and HXTF promoted macrophage formation into the M2 phenotype (Figures 5(a) and 5(b)) and T0070907 promoted macrophage formation into the M1 phenotype (Figures 5(c) and 5(d)).

Compared with the control group, the nuclear translocation level of the RSG group and the HXTF group was significantly increased and the nuclear translocation level of the T0070907 group was unchanged or even lower. And then, in the treatment with HXTF, the nuclear translocation level of the RSG+HXTF group was not significantly different from that of the RSG group, but the nuclear translocation level of T0070907+HXTF was higher than that of the T0070907 group. It is indicated that HXTF could promote nuclear translocation of PPAR-*γ* (Figures 5(e)–5(g)).

**Figure 6:**
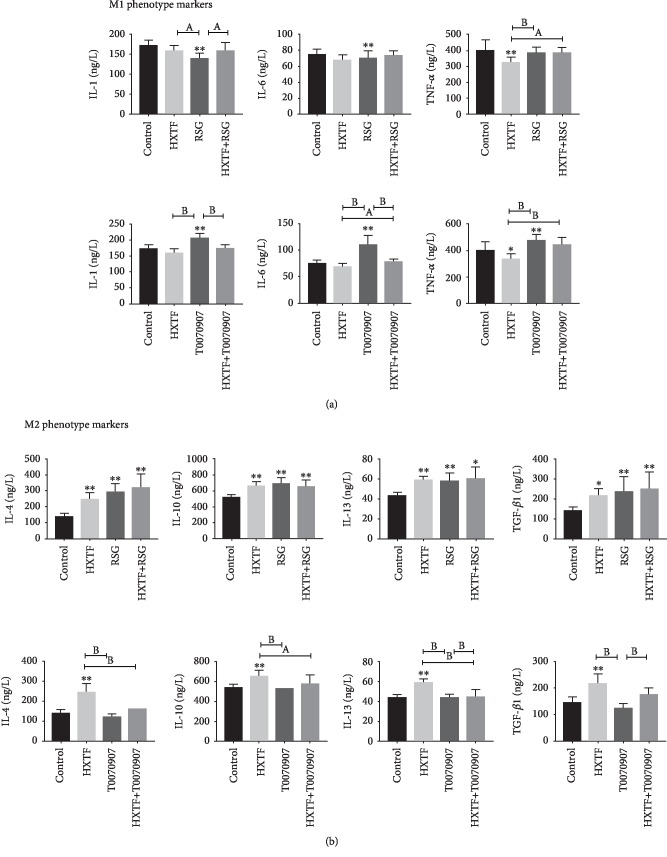
Modulation of PPAR-*γ* activity on M1/M2 markers. RAW264.7 cells were preincubated with either rosiglitazone (RSG) (1 *μ*mol/mL) or T0070907 (1 *μ*mol/mL) for 3 h, followed by combined treatment with/without HuoXueTongFu Formula (HXTF) for 12 h. (a) ELISA analysis of M1 gene marker expression on macrophages. (b) ELISA analysis of M2 gene marker expression on macrophages. All values are expressed as mean ± S.E. (*n* = 3 experiments). ^∗^*p* < 0.05, ^∗∗^*p* < 0.01, ^a^*p* < 0.05, and ^b^*p* < 0.01, comparison of the designated two groups.

The expression of M2 markers was increased significantly, while the expression of M1 markers was decreased after treatment with RSG or HXTF. In contrast, the expression of M1 markers was increased, while the expression of M2 markers was decreased after treatment with T0070907. After macrophage treatment with HXTF, the expression of M1 markers in T0070907 was significantly decreased and the expression of M2 marker was increased (Figure 6).

### 3.7. Effect of PPAR-*γ* Activity on the Macrophage SOCS/JAK2/STAT/PPAR-*γ* Pathway

**Figure 7:**
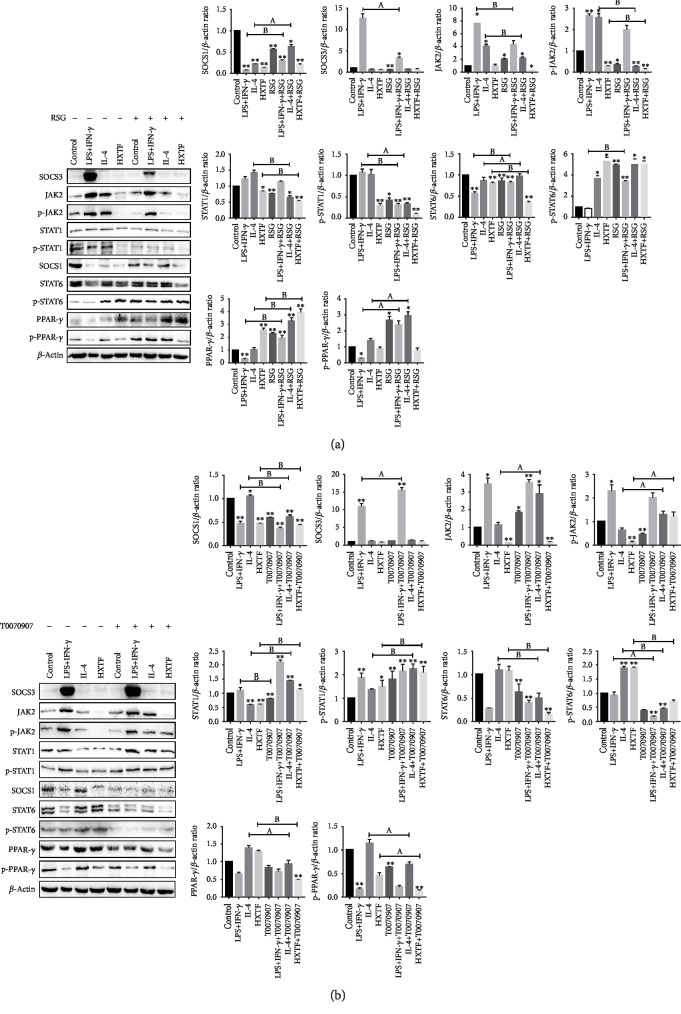
PPAR-*γ* activation on the SOCS/JAK2/STAT/PPAR-*γ* pathway. RAW264.7 cells were preincubated with rosiglitazone (RSG) (1 *μ*mol/mL) or T0070907 (1 *μ*mol/mL) for 3 h, followed by combined treatment with either the control DMEM solution, LPS+IFN-*γ*, IL-4, or HuoXueTongFu Formula (HXTF) treatment for 12 h. The protein expression was assayed by Western blotting. (a) RSG administration on the SOCS/JAK2/STAT/PPAR-*γ* pathway. (b) T0070907 administration on the SOCS/JAK2/STAT/PPAR-*γ* pathway. All values are expressed as mean ± S.E. (*n* = 3 experiments). ^∗^*p* < 0.05, ^∗∗^*p* < 0.01, ^a^*p* < 0.05, and ^b^*p* < 0.01, comparison of the designated two groups.

We have previously shown that PPAR-*γ* activation can tilt macrophages toward the M2 phenotype, and now we want to explore its effect on the SOCS/JAK2/STAT/PPAR-*γ* pathway. We found that the expression of SOCS1, STAT6, PPAR-*γ*, p-STAT6, and p-PPAR-*γ* increased and the expression of SOCS3, JAK2, STAT1, p-JAK2, and p-STAT1 decreased after the use of RSG (Figure 7(a)). At the same time, the use of T0070907 showed the opposite expression (Figure 7(b)). The results suggest that PPAR-*γ* activation inhibits the SOCS3/JAK2/STAT1 pathway and activates the SOCS1/STAT6/PPAR-*γ* pathway in macrophages.

### 3.8. HXTF Reduces the Adhesion Score

**Figure 8:**
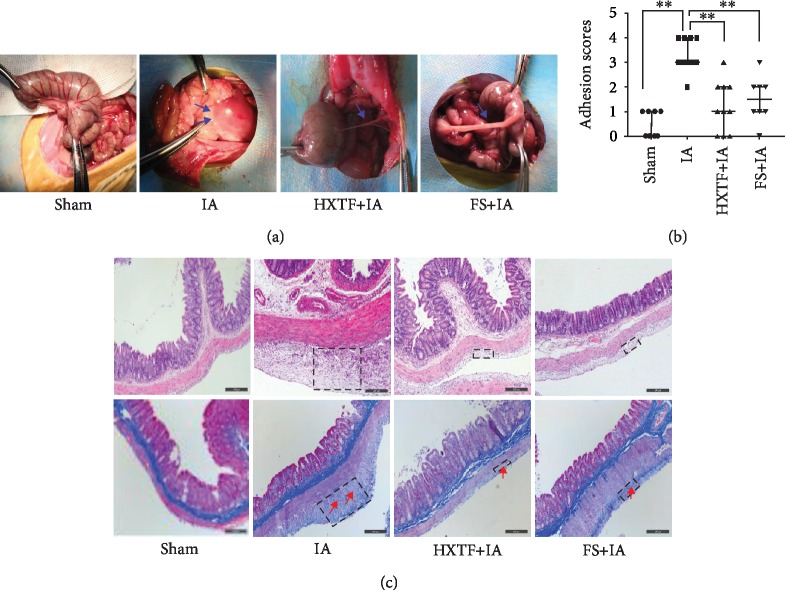
Effect of HuoXueTongFu Formula (HXTF) on reducing adhesion formation. Groups: the sham group, the intraperitoneal adhesion group (IA), the HXTF+IA group, and the fluvastatin (FS)+IA group. (a) Gross observation of adhesion formation. (b) The adhesion score between groups. Data are expressed as the median ± IQR (*n* = 10). ^∗^*p* < 0.05 and ^∗∗^*p* < 0.01, respectively. (c) Effect of HXTF on histological changes of the cecum (HE staining and Masson staining, the black dotted box indicates the adhesion area and the red arrow indicates the collagen deposition. Original magnification, ×100, bar = 200 *μ*m).

Forty rats were successfully anesthetized and operated, but two rats in the FS+IA group died within 48 hours after surgery and were excluded from the study. The remaining 38 surgical rats were normal and entered in the result analysis. Studies have shown that FS can effectively reduce the formation of abdominal adhesion, so we chose FS as the positive control group [23, 24]. Compared to the sham group, the IA group resulted in severe peritoneal adhesions of a higher grade and a higher rate of adhesions. We also found that compared with the IA group, the group treated with HXTF or FS had significantly reduced peritoneal adhesion development rates and adhesion grades (Figure 8(a)). The adhesion score was evaluated as shown in Figure 8(b); the sham group (median, 0.00; IQR, 0.00-1.00) showed significantly a lower adhesion score than the adhesion model animals. The adhesion scores of the HXTF group (median, 1.00; IQR, 0.00-2.00) and FS group (median, 1.5; IQR, 1.00-2.00) were lower than that of the IA group (median, 3.00; IQR, 3.00-4.00). These results indicate that HXTF can significantly reduce adhesion formation.

### 3.9. HXTF Inhibits Inflammation and Collagen Fibre Formation

We used HE staining and Masson staining to observe the changes of cecal structure. As shown in Figure 8(c), no inflammatory cell infiltration and collagen fibres were found in the sham group, and the cecum tended to have a normal structure. In the model group, a large number of inflammatory cells and collagen fibres were observed, accompanied by some muscle fibres, which were connected with the serosal layer. Compared with the model group, the inflammatory cells and the collagen fibres in the HXTF group and FS group were less, and a thinner adhesion area was observed. In conclusion, HXTF can significantly decrease the inflammation and collagen fibre formation.

### 3.10. Effect of HXTF on the SOCS/JAK2/STAT/PPAR-*γ* Pathway in Rats

**Figure 9:**
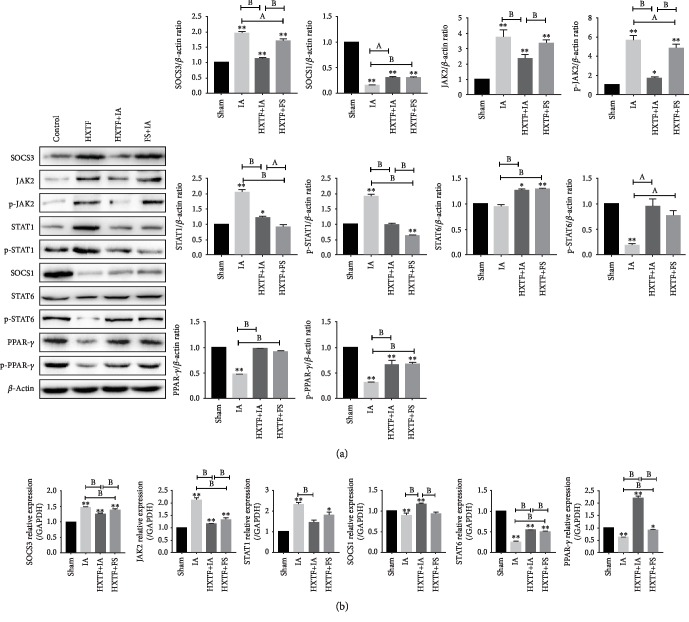
Expression of the SOCS/JAK2/STAT/PPAR-*γ* pathway in intraperitoneal adhesion tissues. (a) Representative Western blot analysis showing SOCS/JAK2/STAT/PPAR-*γ* pathway protein expression levels. (b) Real-time PCR was used to quantify the relative levels of mRNA of SOCS/JAK2/STAT/PPAR-*γ* pathway-related factors. All values are expressed as mean ± S.E. (*n* = 10). ^∗^*p* < 0.05, ^∗∗^*p* < 0.01, ^a^*p* < 0.05, and ^b^*p* < 0.01, comparison of the designated two groups.

We detected the SOCS/JAK2/STAT/PPAR-*γ* pathway-related factors by real-time PCR and Western blot. Compared with the sham group, SOCS3, JAK2, STAT1, p-JAK2, and p-STAT1 were significantly increased in the IA group, while SOCS1, STAT6, PPAR-*γ*, p-STAT6, and p-PPAR-*γ* were significantly decreased. Compared with the IA group, SOCS3, JAK2, STAT1, p-JAK2, and p-STAT1 were significantly decreased in the HXTF+IA and FS+IA groups, while SOCS1, STAT6, PPAR-*γ*, p-STAT6, and p-PPAR-*γ* were significantly increased (Figure 9). In summary, the results show that HXTF has a good inhibitory effect on the SOCS3/JAK2/STAT1 pathway and a good activation effect on the SOCS1/STAT6/PPAR-*γ* pathway in rats.

## 4. Discussion

The peritoneum, after surgery, infection, trauma, or radiation, can cause abdominal tissue injury and inflammation, leading to the formation of pathological “connections” between the omentum, intestinal loops, viscera, and abdominal wall. In this study, HXTF was proven to be effective. We investigated the polarization of HXTF-induced macrophages in vitro and explored the relationship between PPAR-*γ* activity and M1/M2 polarization. We also observed whether HXTF regulates the SOCS/JAK2/STAT/PPAR-*γ* signalling pathway by controlling PPAR-*γ* activity, so as to regulate macrophage polarization and further play an anti-intraperitoneal adhesion role.

Our experiments have shown that hydroxysafflor yellow A, tetrahydropalmatine, kaempferol, aloe-emodin, rhein, emodin, chrysophanol, and emodin methyl ether are the most effective components of HXTF. These active ingredients have been proven to have strong anti-inflammatory properties [25–30]. Hydroxysafflor yellow A is the main active ingredient of Chinese medicine safflower, a natural active ingredient with strong antioxidation and anti-inflammatory functions. It has been reported that hydroxysafflower yellow A can inhibit inflammation by regulating phosphorylation of the JAK2/STAT3 pathway and can reduce fibrosis by activating PPAR-*γ* [31–33]. Kaempferol is a natural flavonoid that effectively inhibits the activation of STAT1 and promotes the activation of STAT6 induced by IL-4 through targeting JAK3 [34, 35]. Emodin and chrysophanol are derivatives of traditional Chinese medicine rhubarb, which can alleviate the LPS-induced inflammatory response of RAW264.7 macrophages and inhibit LPS-induced downregulation of PPAR-*γ* [36–38]. The above reports provide evidence for HXTF to decrease postoperative inflammation and reduce abdominal adhesion.

Inflammation plays an important role in the regulation of the coagulation and fibrinolysis system and is the key mechanism for intraperitoneal adhesion formation [39]. Especially after a peritoneal injury caused by operation and infection, acute inflammation will be triggered first, which will lead to the activation of the coagulation system and collagen deposition. Moderate inflammation can promote wound healing, but excessive inflammation can lead to adhesion formation [40]. Macrophages are innate immune cells that play different roles in regulating inflammation, tissue repair, and other microbial infections [41]. Macrophage polarization is an important mechanism for regulating the inflammatory response, and M1 phenotype macrophages act as cellular mediators for acute and chronic inflammation [42–44], while M2 phenotype macrophages exert anti-inflammatory and prohealing properties and reduce fibrosis as the disease progresses [45, 46]. Therefore, the balance between M1 and M2 is crucial for the healing and remodeling of damaged tissues. The M1 phenotype is stimulated by IFN-*γ*, TNF, and TLR ligands, releasing relatively high levels of proinflammatory cytokines such as IL-1, IL-6, and TNF-*α* [14]. Conversely, the M2 phenotype can be induced by stimulation with IL-4, IL-10, or IL-13, releasing relatively high levels of anti-inflammatory cytokines such as IL-10 and TGF-*β*1 [47, 48]. M1/M2 polarization is a dynamic process that is reversible under physiological and pathological conditions, with heterogeneity and plasticity [49]. As we found in our experiments, macrophages were polarized to the M1 phenotype after stimulation with IFN-*γ* and LPS and were able to produce a variety of proinflammatory cytokines, such as IL-1, IL-6, and TNF-*α*. The expression of inflammatory cytokines was significantly increased, and the expression of anti-inflammatory cytokines such as IL-4, IL-10, and TGF-*β*1 was decreased. However, further HXTF intervention reduced the expression of these proinflammatory factors, enhanced the expression of anti-inflammatory factors, and reversed the polarization trend of M1. Meanwhile, we found that macrophages treated directly with HXTF showed a tendency of M2 polarization similar to IL-4, and the expression of the M2 marker increased.

The JAK/STAT signalling pathway is active in macrophages and is strongly activated after IFN-*γ* stimulation. The dynamic regulation of this signalling pathway is different under steady-state and pathological conditions, and dysregulation of signal transduction can lead to chronic inflammation [13]. JAK2 is an important factor regulating the function of macrophages. It has been found that LPS or IFN-*γ* can induce JAK2 phosphorylation in macrophages, further leading to STAT1 phosphorylation, thereby promoting inflammation [50, 51]. Different SOCS family members play different regulatory roles in the M1/M2 polarization. SOCS1 is strongly upregulated in the M2-polarized environment in vivo and in vitro by blocking JAK tyrosine kinase activity or STAT activation [52, 53]. When the expression of SOCS1 is downregulated, the JAK1/STAT1 pathway is activated, which promotes the polarization of macrophages into the M1 phenotype [54]. However, SOCS3 has shown two opposite effects on macrophage polarization in different studies. One is that the activation of SOCS3 can negatively regulate the JAK2/STAT3 pathway induced by IFN-*γ* to enhance M2 polarization [55, 56]. Another is that SOCS3 is significantly increased in the environment with a large amount of M1 activation in vivo and that targeted reduction of SOCS3 significantly reduces the expression of proinflammatory markers and improves inflammation [57–59]. We support the latter argument through both in vivo and in vitro experiments. We found that SOCS3 is upregulated in both IFN-*γ*+LPS-treated macrophages and the adhesion group of rats and the expression of JAK2 and STAT1 was also increased. The expression of SOCS3, JAK2, and STAT1 is decreased after the intervention of HXTF, while the expression of STAT6 and SOCS1 is increased. After the intervention of HXTF, the expressions of SOCS3, JAK2, and STAT1 were decreased, while the expressions of STAT6 and SOCS1 were increased. The results indicated that HXTF effectively reduces the inflammatory response by inhibiting the SOCS3/JAK2/STAT1 signalling pathway, thus playing a role in the prevention and treatment of intraperitoneal adhesions.

PPAR-*γ*, a transcriptional activator, plays an important role in cell growth, metabolism, apoptosis, and inflammation [60, 61]. PPAR-*γ* is induced by IL-4 and is involved in the activation of M2 macrophages [62]. PPAR-*γ* plays an anti-inflammatory role by inhibiting the activity of inflammatory transcription factors such as activator protein-1 (AP-1), NF-kappa B, and STAT1 [63]. Stat6 is a promoter of PPAR-*γ* transcription, which increases the number of regulatory genes and the intensity of the reaction by promoting DNA binding. Besides, STAT6 interacts with PPAR-*γ* on the promoter of PPAR-*γ* target genes (including Fabp4) to enhance IL-4-induced PPAR-*γ* activity [64]. In the absence PPAR-*γ*, macrophages produce higher levels of inflammation-related metabolites and express a proinflammatory transcriptome [62, 65]. Importantly, local injection of PPAR-*γ* agonists was found to transform macrophages from a proinflammatory M1 phenotype to a prohealing M2 phenotype, reducing inflammation and improving healing [66]. In our study, we stimulated macrophages with RSG, T0070907, and HXTF, respectively. It was observed that the PPAR-*γ* translocation of RSG and HXTF and the expression of the M2 marker were significantly higher than those of the T0070907 group, and the expression of the M1 marker in the T0070907 group was significantly increased. After T0070907+HXTF intervention, it was found that the expression of PPAR-*γ* nuclear translocation and the M2 marker was significantly higher than that of the T0070907 group and the expression of M1 markers was decreased. This indicated that HXTF promotes nuclear translocation of PPAR-*γ* and may regulate M1/M2 polarization by PPAR-*γ*. Meanwhile, our in vitro study confirmed that the expression of SOCS3/JAK2/STAT1 and SOCS1/STAT6/PPAR-*γ* pathways was changed after RSG and T0070907 were given, respectively. The SOCS3/JAK2/STAT1 pathway was inhibited in the RSG group, and the SOCS1/STAT6/PPAR-*γ* pathway was activated. The T0070907 group presented an opposite trend. The above indicated that the SOCS/JAK2/STAT/PPAR-*γ* pathway might be affected by the activation state of PPAR-*γ*. In this process, HXTF had a positive effect on PPAR-*γ* activation.

## 5. Conclusion

This study mainly solves four problems: (1) HXTF can improve postoperative abdominal adhesion, (2) HXTF regulates the polarization of macrophages to M2 and reduces inflammation, (3) HXTF may regulate macrophage polarization through the SOCS/JAK2/STAT/PPAR-*γ* pathway, and (4) the PPAR-*γ* activation state has a regulatory effect on the macrophage SOCS/JAK2/STAT/PPAR-*γ* pathway and affects M1/M2 polarization.

At present, our results show that HXTF can effectively improve the adhesion in rats with abdominal adhesion and reduce the inflammation of macrophages. We still need more research to determine the mechanisms involved in HXTF, but our study provides evidence that Chinese medicine has a regulatory effect on intraperitoneal adhesions in rats and macrophage cells in vitro. This result indicates that it can be used as an option to prevent intraperitoneal adhesions.

## Figures and Tables

**Table 1:** Herbs in HuoXueTongFu Formula.

Chinese name	English name	Latin name	Used part
Da Huang	Chinese rhubarb	Radix et Rhizoma Rhei	Root
Tao Ren	Peach kernel	Semen Persicae	Seed
Yan Hu Suo	Corydalis yanhusuo	Rhizoma Corydalis	Tuber
Lai Fu Zi	Radish seed	Semen Raphani	Seed
Mang Xiao	Glauber's salt	Natrii Sulfas	Crystallization
Hong Hua	Safflower	Flos Carthami	Flower

**Table 2:** Classification for extent and severity of intraperitoneal adhesion.

Grade	Type
0	None
1	Thin, avascular, transparent, easily separated adhesion
2	Weak adhesions, avascular, opaque, lysed with traction
3	Thick, capillaries, opaque, extensive visceral adhesions, extensive visceral adhesions, sharp dissection required
4	Thick, opaque, large vessels, extensive, dense adhesions that involved the adjacent mesentery, intestines, and omentum and extended to the abdominal wall, sharp dissection required

**Table 3:** Primers used for real-time PCR.

Primers	Sequence
R-GAPDH-S	CTGGAGAAACCTGCCAAGTATG
R-GAPDH-A	GGTGGAAGAATGGGAGTTGCT
R-JAK2-S	CAGCAAACTAAAGAAGGCAGGA
R-JAK2-A	TTCTCGCTCAACGGCAAAG
R-Stat1-S	CCTGTGGTACAACATGCTGGTG
R-Stat1-A	TTGGTGACTGACGAAAACTGCC
R-stat6-S	TGCCCTACTTTCTGCCACTGTC
R-stat6-A	ATCCTGGTCTCCCTTACTCGGT
R-Socs1-S	GAGCTGCTGGAGCACTACGT
R-Socs1-A	GGAGTACCGGGTTAAGAGGGA
R-Socs3-S	GGTCACCCACAGCAAGTTTCC
R-Socs3-A	GCACTGGATGCGTAGGTTCTTG
R-PPAR-*γ*-S	CCCTTTACCACGGTTGATTTC
R-PPAR-*γ*-A	CTTCAATCGGATGGTTCTTCG

## Data Availability

The data used to support the findings of this study are available from the corresponding author upon request.

## Supplementary Materials

Supplementary Materials Figure S1: Effect of HuoXueTongFu Formula (HXTF)—medicated serum on the viability of RAW264.7 cells. The group without HXTF-medicated serum used 10% FBS. All values are expressed as mean ± S.E. (*n* = 3 experiments).

## References

[1] Ouaïssi M., Gaujoux S., Veyrie N. (2012). Post-operative adhesions after digestive surgery: their incidence and prevention: review of the literature. *Journal of Visceral Surgery*.

[2] Moris D., Chakedis J., Rahnemai-Azar A. A. (2017). Postoperative abdominal adhesions: clinical significance and advances in prevention and management. *Journal of Gastrointestinal Surgery*.

[3] Attard J.-A. P., MacLean A. R. (2007). Adhesive small bowel obstruction: epidemiology, biology and prevention. *Canadian Journal of Surgery*.

[4] Tingstedt B., Isaksson J., Andersson R. (2007). Long-term follow-up and cost analysis following surgery for small bowel obstruction caused by intra-abdominal adhesions. *The British Journal of Surgery*.

[5] Ray N. F., Denton W. G., Thamer M., Henderson S. C., Perry S. (1998). Abdominal adhesiolysis: inpatient care and expenditures in the United States in 1994. *Journal of the American College of Surgeons*.

[6] Wei G., Chen X., Wang G., Fan L., Wang K., Li X. (2016). Effect of resveratrol on the prevention of intra-abdominal adhesion formation in a rat model. *Cellular Physiology and Biochemistry*.

[7] Hellebrekers B. W. J., Kooistra T. (2011). Pathogenesis of postoperative adhesion formation. *The British Journal of Surgery*.

[8] Hong G. S., Schwandt T., Stein K. (2015). Effects of macrophage-dependent peroxisome proliferator-activated receptor *γ* signalling on adhesion formation after abdominal surgery in an experimental model. *The British Journal of Surgery*.

[9] Shapouri-Moghaddam A., Mohammadian S., Vazini H. (2018). Macrophage plasticity, polarization, and function in health and disease. *Journal of Cellular Physiology*.

[10] Gordon S., Martinez F. O. (2010). Alternative activation of macrophages: mechanism and functions. *Immunity*.

[11] Martinez F. O., Sica A., Mantovani A., Locati M. (2008). Macrophage activation and polarization. *Frontiers in Bioscience*.

[12] ten Broek R. P. G., Stommel M. W. J., Strik C., van Laarhoven C. J. H. M., Keus F., van Goor H. (2014). Benefits and harms of adhesion barriers for abdominal surgery: a systematic review and meta-analysis. *The Lancet*.

[13] Hu X., Chen J., Wang L., Ivashkiv L. B. (2007). Crosstalk among Jak-STAT, Toll-like receptor, and ITAM-dependent pathways in macrophage activation. *Journal of Leukocyte Biology*.

[14] Sica A., Mantovani A. (2012). Macrophage plasticity and polarization: in vivo veritas. *The Journal of Clinical Investigation*.

[15] Yan S., Yue Y. Z., Zeng L. L. (2017). Effects of classical prescriptions of blood-activating and organ-purging formula on immunologic barrier function of intestinal mucosa in rats with postoperative peritoneal adhesion. *Journal of Nanjing University of Traditional Chinese Medicine*.

[16] Yang L. L., Zeng L., Li W. L., Lian X. Y., Gao H. T., Xue K. L. (2017). Influence of Huoxue Tongfu Formula on expression of Nrf2,HO-1,NQO1 and mRNA in rats with postoperative peritoneal adhesion. *Journal of Nanjing University of Traditional Chinese Medicine*.

[17] Zeng L., Qian H. H., Zhao Q. N., Yang J. H. (2010). Huo xue tong fu Fang (HXTFF) in adhesive intestinal obstruction: clinical observation of 56 cases. *Journal of Nanjing University of Traditional Chinese Medicine*.

[18] Harris E. S., Morgan R. F., Rodeheaver G. T. (1995). Analysis of the kinetics of peritoneal adhesion formation in the rat and evaluation of potential antiadhesive agents. *Surgery*.

[19] Üreyen O., Üstuner M. A., Argon A. (2018). The effect of resveratrol and octreotide on peritoneal adhesions in a rat model. *The Malaysian Journal of Pathology*.

[20] Cai X., Hu S., Yu B. (2018). Transglutaminase-catalyzed preparation of crosslinked carboxymethyl chitosan/carboxymethyl cellulose/collagen composite membrane for postsurgical peritoneal adhesion prevention. *Carbohydrate Polymers*.

[21] Morán-Salvador E., Titos E., Rius B. (2013). Cell-specific PPAR*γ* deficiency establishes anti-inflammatory and anti-fibrogenic properties for this nuclear receptor in non-parenchymal liver cells. *Journal of Hepatology*.

[22] Bouhlel M. A., Derudas B., Rigamonti E. (2007). PPAR*γ* activation primes human monocytes into alternative M2 macrophages with anti-inflammatory properties. *Cell Metabolism*.

[23] Hoscan Y., Karabulut Z., Hoscan M. B., Arikan S., Ögüs E., Müderrisoglu H. (2010). Oral fluvastatin reduces the severity of peritoneal adhesions in rats. *Acta Chirurgica Belgica*.

[24] Yue Y., Yan S., Li H., Zong Y., Yue J., Zeng L. (2018). The role of oral fluvastatin on postoperative peritoneal adhesion formation in an experimental rat model. *Acta Chirurgica Belgica*.

[25] Jin M., Sun C. Y., Zang B. X. (2016). Hydroxysafflor yellow A attenuate lipopolysaccharide-induced endothelium inflammatory injury. *Chinese Journal of Integrative Medicine*.

[26] Zhang Y., Sha R., Wang K., Li H., Yan B., Zhou N. (2018). Protective effects of tetrahydropalmatine against ketamine-induced learning and memory injury via antioxidative, anti-inflammatory and anti-apoptotic mechanisms in mice. *Molecular Medicine Reports*.

[27] Yeon M. J., Lee M. H., Kim D. H. (2018). Anti-inflammatory effects of kaempferol on *Helicobacter pylori*-induced inflammation. *Bioscience, Biotechnology, and Biochemistry*.

[28] Zeng H. H., Huang Y. R., Li Z. J., Wang Y., Zhang S. (2018). Effects of emodin on oxidative stress and inflammatory response in rats with acute spinal cord injury. *Zhongguo Zhong Yao Za Zhi*.

[29] Bi F., Chen F., Li Y., Wei A., Cao W. (2018). Klotho preservation by rhein promotes toll-like receptor 4 proteolysis and attenuates lipopolysaccharide-induced acute kidney injury. *Journal of Molecular Medicine (Berlin, Germany)*.

[30] Chae U., Min J. S., Lee H. (2017). Chrysophanol suppresses pro-inflammatory response in microglia *via* regulation of Drp1-dependent mitochondrial fission. *Immunopharmacology and Immunotoxicology*.

[31] Zhang Z. H., Yu L. J., Hui X. C. (2014). Hydroxy-safflor yellow A attenuates A*β*_1-42_-induced inflammation by modulating the JAK2/STAT3/NF-*κ*B pathway. *Brain Research*.

[32] Zhang Z., Wu Z., Zhu X., Hui X., Pan J., Xu Y. (2014). Hydroxy-safflor yellow A inhibits neuroinflammation mediated by A*β*_1–42_ in BV-2 cells. *Neuroscience Letters*.

[33] Wang C. Y., Liu Q., Huang Q. X. (2013). Activation of PPAR*γ* is required for hydroxysafflor yellow A of *Carthamus tinctorius* to attenuate hepatic fibrosis induced by oxidative stress. *Phytomedicine*.

[34] Hämäläinen M., Nieminen R., Vuorela P., Heinonen M., Moilanen E. (2007). Anti-inflammatory effects of flavonoids: genistein, kaempferol, quercetin, and daidzein inhibit STAT-1 and NF-*κ*B activations, whereas flavone, isorhamnetin, naringenin, and pelargonidin inhibit only NF-*κ*B activation along with their inhibitory effect on iNOS expression and NO production in activated macrophages. *Mediators of Inflammation*.

[35] Cortes J. R., Perez-G M., Rivas M. D., Zamorano J. (2007). Kaempferol inhibits IL-4-induced STAT6 activation by specifically targeting JAK3. *Journal of Immunology*.

[36] Zhu T., Zhang W., Feng S. J., Yu H. P. (2016). Emodin suppresses LPS-induced inflammation in RAW264.7 cells through a PPAR*γ*-dependent pathway. *International Immunopharmacology*.

[37] Wen Q., Mei L., Ye S. (2018). Chrysophanol demonstrates anti-inflammatory properties in LPS-primed RAW 264.7 macrophages through activating PPAR-*γ*. *International Immunopharmacology*.

[38] Li A., Liu Y., Zhai L., Wang L., Lin Z., Wang S. (2016). Activating peroxisome proliferator-activated receptors (PPARs): a new sight for chrysophanol to treat paraquat-induced lung injury. *Inflammation*.

[39] Pismensky S. V., Kalzhanov Z. R., Eliseeva M. Y., Kosmas I. P., Mynbaev O. A. (2011). Severe inflammatory reaction induced by peritoneal trauma is the key driving mechanism of postoperative adhesion formation. *BMC Surgery*.

[40] Fu Y., Tsauo J., Sun Y. (2018). Developmental endothelial locus-1 prevents development of peritoneal adhesions in mice. *Biochemical and Biophysical Research Communications*.

[41] Ivanova E. A., Orekhov A. N. (2016). Monocyte activation in immunopathology: cellular test for development of diagnostics and therapy. *Journal of Immunology Research*.

[42] Medzhitov R. (2008). Origin and physiological roles of inflammation. *Nature*.

[43] de las Casas-Engel M., Corbí A. L., Camps J. (2014). Serotonin modulation of macrophage polarization: inflammation and beyond. *Oxidative stress and inflammation in non-communicable diseases - molecular mechanisms and perspectives in therapeutics*.

[44] Fujiwara N., Kobayashi K. (2005). Macrophages in inflammation. *Current Drug Targets. Inflammation and Allergy*.

[45] Cao Q., Wang Y., Zheng D. (2010). IL-10/TGF-*β*–modified macrophages induce regulatory T cells and protect against adriamycin nephrosis. *Journal of the American Society of Nephrology*.

[46] Martinez F. O., Helming L., Gordon S. (2009). Alternative activation of macrophages: an immunologic functional perspective. *Annual Review of Immunology*.

[47] Verreck F. A. W., de Boer T., Langenberg D. M. L. (2004). Human IL-23-producing type 1 macrophages promote but IL-10-producing type 2 macrophages subvert immunity to (myco)bacteria. *Proceedings of the National Academy of Sciences of the United States of America*.

[48] Oliveira M. I., Santos S. G., Oliveira M. J., Torres A. L., Barbosa M. A. (2012). Chitosan drives anti-inflammatory macrophage polarisation and pro-inflammatory dendritic cell stimulation. *European Cells and Materials*.

[49] Gordon S., Pluddemann A. (2013). Tissue macrophage heterogeneity: issues and prospects. *Seminars in Immunopathology*.

[50] Rawlings J. S., Rosler K. M., Harrison D. A. (2004). The JAK/STAT signaling pathway. *Journal of Cell Science*.

[51] Desai H. R., Sivasubramaniyam T., Revelo X. S. (2017). Macrophage JAK2 deficiency protects against high-fat diet-induced inflammation. *Scientific Reports*.

[52] Tang L., Zhang H., Wang C., Li H., Zhang Q., Bai J. (2017). M2A and M2C macrophage subsets ameliorate inflammation and fibroproliferation in acute lung injury through interleukin 10 pathway. *Shock*.

[53] Whyte C. S., Bishop E. T., Rückerl D. (2011). Suppressor of cytokine signaling (SOCS)1 is a key determinant of differential macrophage activation and function. *Journal of Leukocyte Biology*.

[54] Morris R., Kershaw N. J., Babon J. J. (2018). The molecular details of cytokine signaling via the JAK/STAT pathway. *Protein Science*.

[55] Peng H. Y., Cheng Y. C., Hsu Y. M. (2016). MPT0B098, a microtubule inhibitor, suppresses JAK2/STAT3 signaling pathway through modulation of SOCS3 stability in oral squamous cell carcinoma. *PLoS One*.

[56] Yu T., Zuo Y., Cai R. (2017). SENP1 regulates IFN-*γ*-STAT1 signaling through STAT3-SOCS3 negative feedback loop. *Journal of Molecular Cell Biology*.

[57] Liu Y., Stewart K. N., Bishop E. (2008). Unique expression of suppressor of cytokine signaling 3 is essential for classical macrophage activation in rodents in vitro and in vivo. *Journal of Immunology*.

[58] Pal S., Nath P., Biswas S., Mukherjee U., Maitra S. (2019). Nonylphenol attenuates SOCS3 expression and M1 polarization in lipopolysaccharide-treated rat splenic macrophages. *Ecotoxicology and Environmental Safety*.

[59] Gordon P., Okai B., Hoare J. I., Erwig L. P., Wilson H. M. (2016). SOCS3 is a modulator of human macrophage phagocytosis. *Journal of Leukocyte Biology*.

[60] Semple R. K., Chatterjee V. K., O'Rahilly S. (2006). PPAR*γ* and human metabolic disease. *The Journal of Clinical Investigation*.

[61] Kersten S., Desvergne B., Wahli W. (2000). Roles of PPARs in health and disease. *Nature*.

[62] Nelson V. L., Nguyen H. C. B., Garcìa-Cañaveras J. C. (2018). PPAR*γ* is a nexus controlling alternative activation of macrophages via glutamine metabolism. *Genes & Development*.

[63] Jacobi D., Stanya K., Lee C. H. (2012). Adipose tissue signaling by nuclear receptors in metabolic complications of obesity. *Adipocytes*.

[64] Szanto A., Balint B. L., Nagy Z. S. (2010). STAT6 transcription factor is a facilitator of the nuclear receptor PPAR*γ*-regulated gene expression in macrophages and dendritic cells. *Immunity*.

[65] Odegaard J. I., Ricardo-Gonzalez R. R., Goforth M. H. (2007). Macrophage-specific PPAR*γ* controls alternative activation and improves insulin resistance. *Nature*.

[66] Mirza R. E., Fang M. M., Novak M. L. (2015). Macrophage PPAR*γ* and impaired wound healing in type 2 diabetes. *The Journal of Pathology*.

