# Supplementary material for: HuoXueTongFu Formula Alleviates Intraperitoneal Adhesion by Regulating Macrophage Polarization and the SOCS/JAK2/STAT/PPAR-γ Signalling Pathway
Source: Mediators Inflamm. 2019 Oct 21;2019:1769374. doi: 10.1155/2019/1769374 (PMC6854253; doi:10.1155/2019/1769374)
Supplement: Supplementary Materials — Figure S1: Effect of HuoXueTongFu Formula (HXTF)—medicated serum on the viability of RAW264.7 cells. The group without HXTF-medicated serum used 10% FBS. All values are expressed as mean ± S.E. (n = 3 experiments). [file 1769374.f1.pdf]

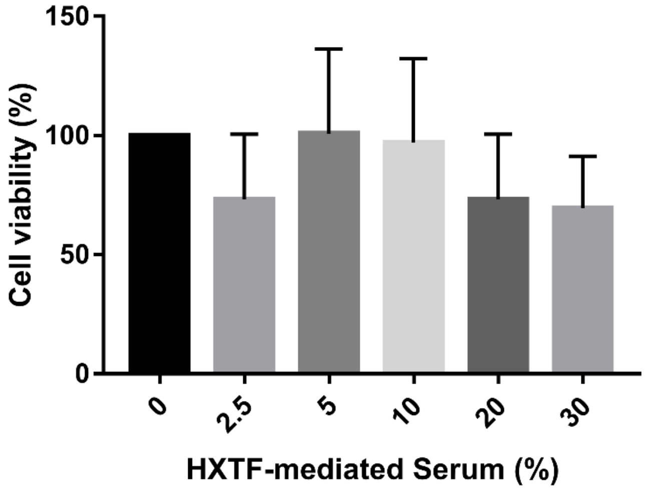

Figure S1: Effect of HuoXueTongFu Formula (HXTF) - medicated serum on the viability of RAW264.7 cells. Group without HXTF-medicated serum used 10% FBS. All values are expressed as mean ± S.E. (n=3 experiments)
